# Criteria for Site Selection of Temporary Shelters after Earthquakes: a Delphi Panel

**DOI:** 10.1371/currents.dis.07ae4415115b4b3d71f99ba8b304b807

**Published:** 2015-11-23

**Authors:** Ahmad Soltani, Ali Ardalan, Ali Darvishi Boloorani, AliAkbar Haghdoost, Mohammad Javad Hosseinzadeh-Attar

**Affiliations:** Department of Disaster Public Health, School of Public Health, Tehran University of Medical Sciences, Tehran, Iran; Disaster and Emergency Health Academy, National Institute of Health Research, School of Public Health, Tehran University of Medical Sciences, Tehran, Iran; Harvard Humanitarian Initiative, Harvard University, Cambridge, USA; Research Center for Modeling in Health, Institute for Future Studies in Health, Kerman University of Medical Sciences, Kerman, Iran; Department of Clinical Nutrition, School of Nutritional Sciences and Dietetics, Tehran University of Medical Sciences, Tehran, Iran

## Abstract

Introduction: After a devastating earthquake, the site selection for the sheltering of earthquake victims is an important task. In order to generate a list of appropriate criteria for deciding on temporary sheltering site selection, we systematically combined the experience of experts and the findings of published documents in this study.

Methods: Having explored published papers, we generated a list of criteria for the selection of the best location for temporary sheltering. In the next step, all criteria were presented to a group of experts in Iran and after a scientific discussion, the list was updated. In the last step, the final list of criteria was developed using the Delphi method in three rounds.

Results: Based on our previous systematic review, 27 criteria were presented for sheltering site selection. Expert interviews added 12 more items to them. The Delphi process approved 21 criteria of all proposed ones. These items then grouped into four categories: land suitability, socio-cultural considerations, service availability and disaster risk reduction.

Discussion: After an earthquake, our list of criteria may help the disaster team to select the best locations for temporary sheltering with minimum confusion. The consent of the earthquake victims and cost reduction of the operation would be the minimum benefits of using the appropriate criteria. These criteria also could be used by researchers to make objective and reproducible assessments of temporary sheltering site selection. Key words: Criteria, Earthquake, Model, Site selection, Temporary shelter,

## Introduction

Destruction of houses, whether complete or partial is a consequence of earthquakes which results in homelessness. In the past 10 years, up to 9 million people became homeless worldwide because of earthquakes.^1^

Sheltering, an essential phase in response to earthquakes, refers to the activity of staying in a place after the event even though daily routines are suspended. After destructive earthquakes, this process can last weeks to months and should be considered as one component of a defined long-term strategy.^2 ^^,^ 3 In the United States, this strategy consists of four phases: emergency sheltering, temporary sheltering, temporary housing and permanent housing. Unlike housing which denotes the return to normal daily activities, sheltering is a transitional process. Emergency sheltering is usually designed for only one or two days after the event whereas temporary sheltering is for a longer period after the disaster. This period usually lasts for weeks or months.^3^^,^ 4

In some communities, emergency sheltering is not limited to one or two days and may be used for several weeks. In this situation, emergency sheltering refers to both emergency and temporary sheltering.^2^^,^ 5 ^,^ 6 Moreover in some situation; because of delay in recovery phase, this transitional phase can last longer and temporary shelters would be used as temporary houses or long term settlements. Anyway as an emergency situation and in combination with other earthquake consequences, it is important to shelter earthquake victims properly as soon as possible.

Temporary sheltering should provide at the very least residence, security and dignity for earthquake victims.^2^^,^ 7 So, it needs to be planned in appropriate locations to decrease victims’ problems as far as possible. Such planning needs to define related and proper criteria and should be done in the preparedness phase. Having no plan or selecting inappropriate sites could lead to undesirable consequences such as resistance to acceptance of the site. Lack of safety, delays linked to the procurement of shelters, finding sites, lack of organizational services, subsequent secondary disasters and disease, reinforcement/replacement actions, social problems and cultural or climatic inappropriateness are some of these consequences.^8^^,^ 9 ^,^ 10

There are some recommendations for site selection in the disaster sheltering guidelines, but few of these refer specifically to earthquakes. Also, the available recommendations are not presented on the basis of a shelter strategy planning; a defect which has been commented on in some evaluations.^8^ Moreover, the lack of a definitive list of sheltering site selection criteria is a barrier to the advancement of this process. It's difficult for investigators to evaluate this process in an actual earthquake or compare it with the others effectively.

Most studies on sheltering are descriptions of the events and their problems. They have evaluated the process of camp management and not site selection. However it seems that most of the managerial defects are rooted in the site selection procedure.^8^ Defining a practical standard for this process could help the managers to determine objectively if the operation has been carried out according to the criteria or not.

We determined some presented criteria for sheltering site selection in our previous systematic review. In this study we aimed to define essential criteria which are practical for temporary sheltering site selection after earthquakes. Our findings will help the emergency planners to settle the homeless people in safe and acceptable shelters after the earthquakes. These criteria can be used in developing suitable tools for evaluating temporary shelter site selection after earthquakes.

## Methods

This is a mixed method study which was conducted in three phases. We considered the “temporary phase” of sheltering after earthquakes as our main purpose in order to define the essential site selection criteria.

The results of our previous systematic review of sheltering site selection criteria after earthquakes was used as the basis for a semi-structured key informant interview.^10^ Nine national experts with experience in sheltering were selected for interview in order to discuss the criteria for temporary sheltering site selection. All of them had proven experience in response to earthquake at the national or international levels. They had participated in setting up and management of temporary shelters in previous large earthquakes. They were asked to discuss the essential criteria of temporary sheltering site selection. After each interview, new criteria were added to the initial list. The interviews continued until no new criteria were suggested.

Then a modified Delphi process was used to ask the opinions of some experts about the completed list.^11^ Thirty experts and academics who had experience and knowledge about the response to earthquakes at international, national or local levels were chosen for this phase (Table 1). Academic participants were some faculty staffs who had publications in the field of earthquake and disaster management. They also had experience in response to earthquakes. They were architect, physician, planner or engineer. Other participants were relief experts from Iranian Red Crescent society who had participated in setting up or management of camps in previous earthquakes.

Each participant was sent an e-mail describing the project and inviting him to participate on the panel. In the first Delphi round, the panelists read the completed list and were asked if the proposed criteria are essential for decision making in sheltering site selection after earthquakes or not. The participants were also asked to modify or remove existing criteria or suggest any new criterion.

After the first round, the list was edited on the base of participant feedback. For any modification, the proposed comment was described in the edited list. The edited list was used in the second round and the participants were again asked to send their comments on the list and its modification.

Participant feedback from the second round was assessed by the study researchers and the list was edited where there was at least 80% agreement on the comments. Also, related criteria were classified into four main categories. This revised list was used for third Delphi round. We asked the participants to comment on the list and our categorization. We decided to continue the rounds to reach at least 80% agreement for the selected criteria and their categorization.^11^

**Table 1 – Participants of the modified Delphi process:** 

Perspective	Years of experience	Number of completed round 1	Number of completed round 2	Number of completed round 3
Academic	8 – 15	9	8	8
	16 – 25	3	3	3
	>25	0	0	0
Relief expert	8 – 15	9	8	8
	16 – 25	4	4	4
	>25	5	5	5

## Results

Twenty-seven criteria for sheltering site selection after earthquakes were defined in our previous systematic review.^10^ Expert interviews added at least twelve criteria to this list (Table 2).

Thirty local and national experts, who had well-established experience in response to earthquakes, were invited to participate in our modified Delphi process (Table 1). All of them responded to the first round of the process.

Reviewing proposed comments represented an overlap between some criteria. Some others could be considered after proposing some suitable sites based on other criteria. Two criteria were considered to be more appropriate for emergency sheltering (Table 2).

**Table 2 - Delphi participant comments on proposed criteria for Site Selection of Temporary Shelters after Earthquakes:** 

Criteria	References	Comments of participants
Accessibility	Systematic review, Expert interview	Suitable criterion for temporary sheltering site selection
Suitable size	Systematic review, Expert interview	Could be considered after proposing some suitable sites based on other criteria
Proximity to homes of affected people	Systematic review, Expert interview	Suitable criterion for temporary sheltering site selection
Infrastructure conditions	Systematic review, Expert interview	Suitable criterion for temporary sheltering site selection but needs to be modified
Land drainage	Systematic review, Expert interview	Synonymous with Soil permeability
Soil permeability	Systematic review	Suitable criterion for temporary sheltering site selection
Physical layout and periphery configuration	Systematic review	Synonymous with Accessibility
Suitable distance from hazardous areas	Systematic review, Expert interview	Suitable criterion for temporary sheltering site selection but needs to be modified
Geological hazards	Systematic review, Expert interview	Suitable criterion for temporary sheltering site selection
Land slope	Systematic review, Expert interview	Suitable criterion for temporary sheltering site selection
Elevation	Systematic review	Similar for all sites in the affected region / Could be considered in architectural process
Building protection standards	Systematic review, Expert interview	Suitable criterion for emergency sheltering site selection
Early warning availability	Systematic review	Could be considered in architectural or managerial processes / Suitable criterion for emergency sheltering site selection
Water supply	Systematic review, Expert interview	Suitable criterion for temporary sheltering site selection
Suitable distance from medical centers	Systematic review, Expert interview	Suitable criterion for temporary sheltering site selection
Proximity to relief services	Systematic review, Expert interview	Suitable criterion for temporary sheltering site selection
Communication service	Systematic review, Expert interview	Suitable criterion for temporary sheltering site selection
Security and protection	Systematic review, Expert interview	Suitable criterion for temporary sheltering site selection
Economic considerations	Systematic review	Could be considered after proposing some suitable sites based on other criteria
Land ownership	Systematic review, Expert interview	Suitable criterion for temporary sheltering site selection
Previous land use	Systematic review, Expert interview	Suitable criterion for temporary sheltering site selection
Environmental consideration	Systematic review	Suitable criterion for temporary sheltering site selection
Ecological recovery	Systematic review, Expert interview	Suitable criterion for temporary sheltering site selection but needs to be modified
Vegetation	Systematic review, Expert interview	Suitable criterion for temporary sheltering site selection
Scope for agriculture	Systematic review, Expert interview	Need not be considered for all communities
Culture, tradition and composition of population groups	Systematic review, Expert interview	Synonymous with Public opinion
Public opinion	Systematic review	Could be considered in architectural or managerial processes/ Suitable criterion for temporary sheltering site selection but needs to be modified
Proximity to road networks	Expert interview	Synonymous with Accessibility
Suitable distance from secondary hazards	Expert interview	Suitable criterion for temporary sheltering site selection
Suitable distance from polluting industries	Expert interview	Suitable criterion for temporary sheltering site selection
Cultural heritage considerations	Expert interview	Suitable criterion for temporary sheltering site selection
Dominant wind direction	Expert interview	Similar for all sites in the affected region/ Could be considered in architectural process
Precipitation	Expert interview	Similar for all sites in the affected region / Could be considered in architectural process
Number of affected	Expert interview	Could be considered after proposing some suitable sites based on other criteria / Could be considered in architectural or managerial processes
Season	Expert interview	Similar for all sites in the affected region/ Could be considered in architectural or managerial processes
Predicted time for sheltering	Expert interview	Could be considered after proposing some suitable sites based on other criteria / Could be considered in architectural or managerial processes
Fields for keeping livestock	Expert interview	Need not be considered for all communities
Availability of local materials	Expert interview	Could be considered in architectural process
Shelter type and model	Expert interview	Could be considered in architectural or managerial processes

Scope for agriculture and fields for keeping livestock are two criteria used for some rural regions, where site selection for sheltering tends not be as complicated as for urban ones. In such regions, relief teams rarely have a problem in site selection.

Some criteria could be categorized as part of the architectural or managerial process. These processes should be separated from the site selection process. Shelter type and model, expected time for response and recovery phases and availability of local materials were considered to belong to this group. Even though these criteria could affect the site selection process, some other criteria could cover their effects (Table 2).

Elevation, dominant wind direction, precipitation, considering the season and weather conditions are usually similar for an affected region. Also, they could be managed in the architectural or managerial process.

Reviewing participants’ comments in this round led to a modification of the list to 21 criteria. In the second round, we sent the participants our revised list and explained the feedback from the first round and the changes to the criteria.

Twenty-eight of them responded. The main feedback was the need for revision of four criteria. These were: infrastructure conditions, suitable distance from hazardous areas, environmental considerations and public opinion. We also classified all selected criteria into four main categories (Table 3). These categories were: land suitability, socio-cultural considerations, service availability and disaster risk reduction. Categorized criteria were sent to the participants as the third round of the Delphi. There were few comments on the final list and we had achieved the desired expert consensus (89.26%).

**Table 3 - The modified Delphi Results for Site Selection Criteria of Temporary Shelters after Earthquakes:** 

Main Category	Criteria	Definition
Land suitability	Soil permeability	Swift absorption of surface water by the soil and drainage of surface water and sewage is a key criterion especially where water is readily available. It also influences the effectiveness of pit latrines.
	Land slope	Land slopes steeper than 25% are considered to have a high risk of geo-hazards. Those at 2 – 8% are regarded as stable and secure.
	Vegetation	The sites should provide sufficient ground cover for vegetation.Bushes, grass and trees, for example; supply shade and reduce dust and erosion.
	Land ownership	Ownership and usage rights of each shelter area should be determined.
	Ecological considerations	The site should not be located near areas that are ecologically or environmentally protected or fragile.
	Cultural heritage considerations	The site should not be located near areas that are culturally protected or fragile.
Socio-cultural considerations	Proximity to homes of affected people	Shelters should be evenly distributed so that earthquake victims can arrive there quickly.
	Previous land use	Some types of pre-earthquake land use could affect the health of people or their willingness to live there.
	Compatible neighboring land use	Shelters should be far away from areas which are unpleasant or insalubrious such as prisons, mortuaries, cemeteries, slaughterhouses and chemical factories.
Service availability	Accessibility	Shelters should be evenly distributed so that earthquake victims can enter, exit and receive relief services quickly. Proximity to road networks is important.
	Water supply	Shelters should have water facilities which can provide drinking water, domestic water and fire water.
	Medical services	Shelters should be able to provide medical services. Therefore, such a site should be located as near as possible to medical centers.
	Relief services	Shelters should be evenly distributed so that earthquake victims can receive relief items and services such as food, tents, blankets, water and have access for the fire brigade.
	Security and protection	It is recommended that earthquake victims be settled at a convenient distance from police stations and military installations to ensure their protection and security.
	Electrical networks	It is recommended that the sites be at a reasonable distance from electrical networks to ensure supply of lighting and power needs.
	Communication services	Deployment of communication facilities such as telephones and radios etc.
Disaster risk reduction	Geological hazards	Shelters should be situated away from seismic active fault, landslide, collapse, debris flow, soil liquefaction and ground depression.
	Hydro- meteorological hazards	Shelters should not be situated near riverbeds, waterways, watercourses and flood-sensitive areas.
	Environmental diseases	Shelters should not be situated near sewage outlets, swamps, landfills, aviculture, dairies, stables, waste water treatment facilities, etc.
	Secondary hazards	Shelters should be sitesd far away from any potential dangers, such as tall buildings, flammable and explosive substances, hazardous chemicals, radioactive substances, high voltage transmission lines, airports, railways, highways and every possible secondary hazard.
	Polluting industries	Shelters should be sited far away from any polluting industry such as refineries, factories, etc.

## Discussion

Emergency sheltering provides some primary needs for affected people and differs with temporary sheltering and housing with the aim of returning to normal daily activities.Theses services should be provided for homeless people based on appropriate and applicable criteria.^2^^,^ 3 ^,^ 7 We envisaged temporary sheltering site selection after earthquakes as our main objective in this study. This objective would cover also cases with lasting transitional phase and using temporary shelters as long term settlements.

Despite housing, sheltering is usually done rapidly after the events, and mainly based on individual experiences.^9^^,^ 10 It is very challenging to consider all the essential criteria in such situations. Making a decision when working against the clock after the event is an important drawback in relief operations and usually leads to a higher workload and a budget burden for shelter agencies.^8^^,^ 10 The absence of a preparedness plan, lack of integration by means of a comprehensive strategy and forcing the disaster relief teams to do something for the affected people are some of the reasons for such decisions.

There is no report comparing earthquake and other disasters as regards sheltering site selection. It seems that due to some characteristics such as duration and type of response and recovery phases, sheltering after an earthquakes differs from other disaster situations. We approached our work specifically from an earthquake perspective considering a situation with long-term reconstruction. The planners are faced with massive destruction, infrastructure damages, large debris and probability of aftershocks and secondary disasters.

There are few papers with this approach and most of related papers have described the problems or presented models for shelter site selection based on the available criteria for their defined region.^9^^,^ 10 Such criteria might not be applicable in all communities or situations and their limitations make them useless in other regions or situations. Therefore, our proposed criteria could be a good subject to be used and criticized by other experts.

Having several different criteria makes the decision difficult and almost impossible, especially when they are descriptive and abstract.^12^^,^ 13 Reducing the criteria and focusing on those which are more important and concrete would make the process more acceptable and easier. We aimed to define the related criteria for temporary sheltering site selection, and considered at least three principles: as few criteria as possible; criteria that were independent of each other; and criteria that were quantifiable in estimation.^12^

There are some differences between rural and urban communities in sheltering after the earthquakes.^14^ In rural regions, because of the type of houses, population density, life style and some considerations in relief operation, it is recommended to shelter the affected people near their homes. In urban areas, the strategy usually changes to setting up camps which requires proper site selection. We speculate that the identified criteria could be easily obtained for all regions and be used in developing relevant models or decision support systems for the site selection of camps.

Thirty-nine criteria were presented to be used in this process. Reviewing these criteria showed a similarity between some of them. Indeed, some of these criteria have be**e**n labeled differently in different papers. Applying expert opinion in our review reduced them to 35 criteria. This means we needed to provide a precise definition of the criteria. We limited our findings to temporary sheltering which is only one stage of a long-term strategy in the settlement of affected people. Some of the proposed criteria are mainly used in the emergency phase of sheltering, so we needed to define the shelter strategies and related terminology. Such a definition is a prerequisite for proposing each specific model or a protocol for sheltering.^8^

Some criteria could be used separately before or after assessing the others. Estimating the suitable size is an important criterion which correlates with the number of affected people. We can select our suitable size from a number of proposed sites which have been suggested based on the other criteria. So it is not necessary to use this criterion directly in site selection. This is also true for economic considerations and predicted time for sheltering. Some others such as elevation, precipitation and dominant wind direction are similar for all sites in the affected region.

Some important criteria could not be directly used in site selection. Public opinion belongs to this group. Three criteria in the socio-cultural considerations category cover public opinion. Some relevant issues such as proximity to workplace and educational facilities would be covered by these criteria. Some others could be considered in the architectural or managerial processes of sheltering rather than the site selection. Facilities such as child friendly spaces are good examples which would be considered in designing the camp. So it is important to distinguish site-related subjects from the others.

Our selected criteria as a comprehensive package, could be considered by relief teams in their operations. Also they could be weighted in each community and be used in models to propose suitable sites for sheltering affected people.^12^ Therefore, with respect to the priorities of the community, some criteria would give a minimum weight or raise the weight for them.

Categorizing the criteria provides an overview of the main requirements for decision making in this field. These requirements are land characteristics, socio-cultural considerations, availability of services and disaster risk reduction.

Using the right criteria would reduce decision making errors and save time. It also makes the process more concrete. So, the process will be performed, evaluated and improved by using defined criteria. We propose the design of some studies to evaluate site selection for temporary shelters in the recent earthquakes on the basis of our presented criteria. Such studies could show the probable limitations of using these criteria. We also suggest future study to provide the weights of selected criteria for our community and using them in proposing suitable sites for temporary sheltering in vulnerable cities.

Our findings were developed by a panel of local and national experts who live and work in an earthquake-prone country and the results would be applicable in national guidelines and models. Maybe expanding the panel to include a variety of other international experts and asking them to share their experience would make it even more appropriate as a proposal for international guidelines.

## Conclusion

This study developed a list of criteria which could be used in selecting proper sites for temporary sheltering after an earthquake. Community planners could use these criteria in related models or systems to choose suitable sites for emergency situations. Developing and applying such models should be done in the preparedness phase.

The consent of the affected people and cost reduction of the operation would be the minimum benefits of such an approach. This approach also allows the researchers to start making objective and reproducible assessments of sheltering site selection and helps to improve the outcome ultimately.

## Corresponding author

Ali Ardalan, MD, PhD. School of Public Health, International Campus, Tehran University of Medical Sciences ; Harvard Humanitarian Initiative, Harvard University.

Email: aardalan@tums.ac.ir, ardalan@hsph.harvard.edu

## Competing Interests

The authors have no competing interests.
